# A Combined Experimental and Computational Study of Vam3, a Derivative of Resveratrol, and Syk Interaction

**DOI:** 10.3390/ijms150917188

**Published:** 2014-09-25

**Authors:** Ming Jiang, Renping Liu, Ying Chen, Qisheng Zheng, Saijun Fan, Peixun Liu

**Affiliations:** 1 Key Lab of Tianjin Molecular Nuclear Medicine, Institute of Radiation Medicine, Peking Union Medical College, Chinese Academy of Medical Sciences, Tianjin 300192, China; E-Mails: jiangmingpumc@126.com (M.J.); chenying_tj@hotmail.com (Y.C.); qishengzheng@126.com (Q.Z.); 2 Medical Experiment Education Department, Medical College of Nanchang University, Nanchang 330031, China; E-Mail: sirlrp@163.com

**Keywords:** Vam3, Syk, docking, molecular dynamics simulation, Syk inhibitor

## Abstract

Spleen tyrosine kinase (Syk) plays an indispensable role through preliminary extracellular antigen-induced crosslinking of Fc receptor (FcR) in the pathogenesis of autoimmune disorders, such as rheumatoid arthritis. In this study, we identify Vam3, a dimeric derivative of resveratrol isolated from grapes, as an ATP-competitive inhibitor of Syk with an IC_50_ of 62.95 nM in an *in vitro* kinase assay. Moreover, docking and molecular dynamics simulation approaches were performed to get more detailed information about the binding mode of Vam3 and Syk. The results show that 11b-OH on ring-C and 4b-OH on ring-D could form two hydrogen bonds with Glu449 and Phe382 of Syk, respectively. In addition, arene-cation interaction between ring-D of Vam3 and Lys402 of Syk was also observed. These results indicate that ring-C and D play an essential role in Vam3–Syk interaction. Our studies may be helpful in the structural optimization of Vam3, and also aid the design of novel Syk inhibitors in the future.

## 1. Introduction

Allergic and autoimmune disorders share significant functional overlap in the biologic pathways responsible for the activation of signal transduction events leading to production of numerous proinflammatory factors involved in disease initiation and progression [1]. Given the reciprocal connections in these mechanistic pathways, it would be advantageous to target strategic master regulators with novel therapeutics to treat allergic and autoimmune diseases. One such crucial regulator is spleen tyrosine kinase (Syk). Syk is a cytosolic non-receptor tyrosine kinase. It serves as a key mediator of B-cell receptor and Fc receptor mediated signaling in inflammatory cells such as B cells, mast cells, macrophages, dendritic cells, and neutrophils and is involved in bone resorption by osteoclasts [2,3,4]. Activation of Syk occurs through preliminary extracellular antigen-induced crosslinking of FcεR1 and FcγRs I, IIA, and IIIA. Upon abnormal activation, Syk, a master upstream regulator of signal transduction, propagates downstream signaling molecules, resulting in initiation of disease [3]. Furthermore, in specific contexts, uncontrolled activation of B cell receptor (BCR) signaling via Syk would lead to development of lymphomas and leukemia. Murine studies have shown that Syk expression is required for the survival of Non-Hodgkin Lymphomas-like (NHL-like) tumors *in vitro*. Pharmacologic inhibition of Syk induced apoptosis in murine B-cell lymphomas *in vitro* and resulted in regression of NHL-like B-cell lymphomas [1,5].

Full-length Syk is composed of two *N*-terminal Src homology 2 (SH2) domains followed by an interdomain linker and a *C*-terminal kinase domain [6]. The tandem SH2 (tSH2) module is also separated by an inter-SH2 linker and serves as a docking platform for immune receptor tyrosine-based activating motifs (ITAMs) which are displayed at the cytosolic side of the plasma membrane [7,8]. The truncated kinase domain of Syk (Syk-KD), which contains an ATP-binding pocket shows significant catalytic activity and has been extensively used for inhibitor design [9].

**Figure 1:**
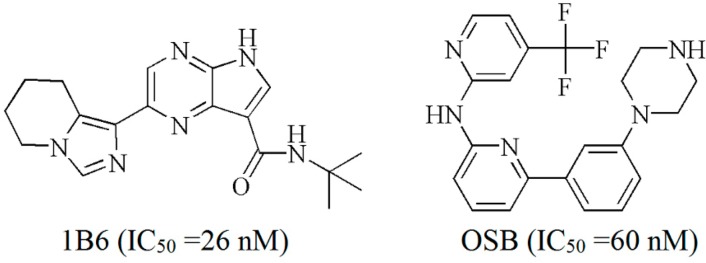
Structures of 1B6 and OSB.

In recent years, a number of Syk inhibitors have been discovered for treatment of autoimmune, allergic and autoinflammatory diseases and most of them are ATP-competitive Syk inhibitors [3,10,11,12]. Several research groups have independently reported the crystal structures of the Syk catalytic domain, co-crystallized and ligand soaked with small molecule inhibitors [13,14,15,16]. For example, as shown in Figure 1, OSB and 1B6 are two ATP-competitive inhibitors of Syk with an IC_50_ of 60 and 26 nM, respectively. The crystal structures of OSB and 1B6 with the catalytic domain of Syk were reported by Marcos Castillo *et al.* and Fernando Padilla *et al.*, respectively [10,12].

**Figure 2:**
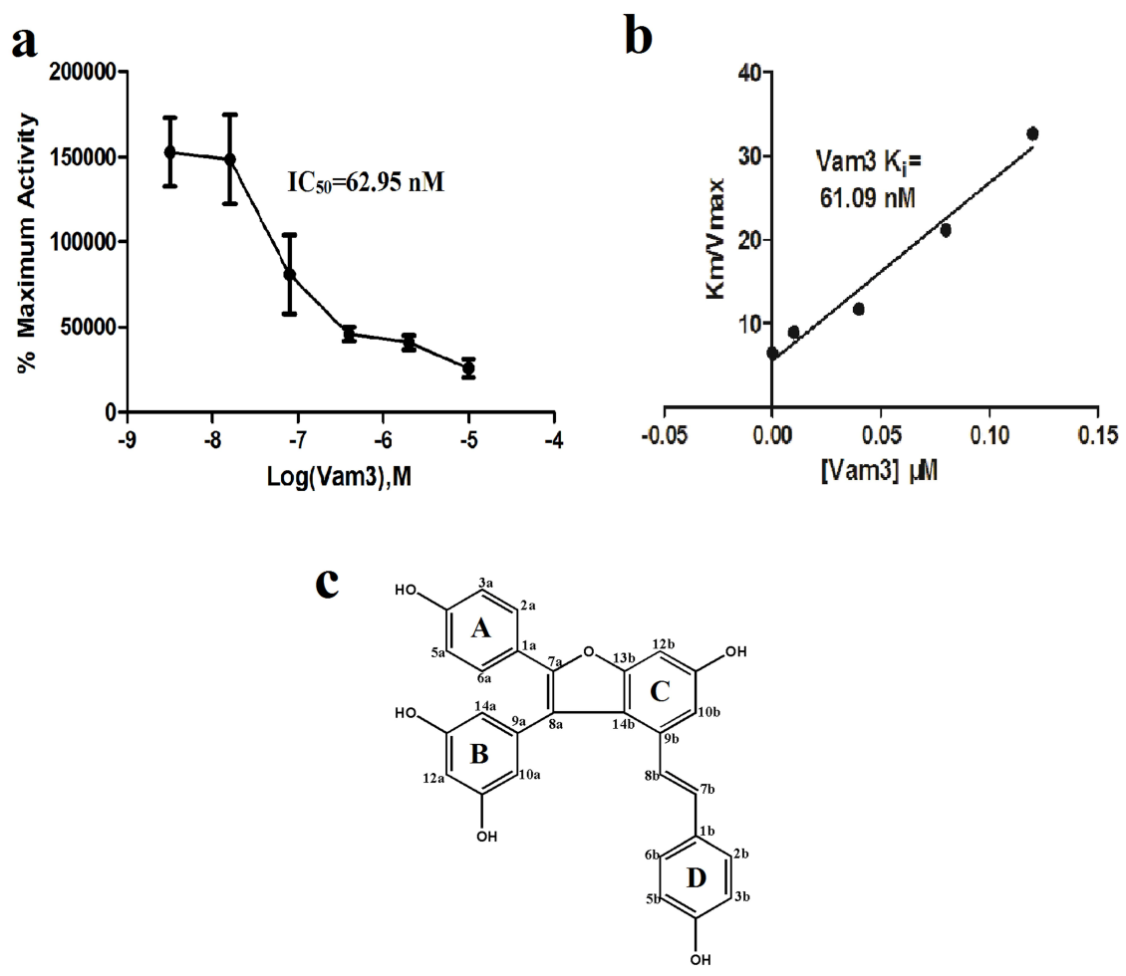
(**a**) IC_50_ determination of Vam3 with recombination Syk protein; (**b**) Ki determination of Vam3 with recombination Syk protein; (**c**) Chemical structure of Vam3.

Vam3 (amurensis H), a resveratrol dimer, was first isolated from ethanol extracts of *Vitis amurensis* Rupr as a secondary natural product. Previous studies indicated that Vam3 has anti-inflammatory effects, including alleviate the asthmatic inflammation in asthmatic mice and decrease cigarette smoke-induced autophagy in human bronchial epithelial cells [17,18]. However, the molecular basis by which Vam3 inhibits inflammation is not clear. In this study, we identified Vam3 as a potent ATP-competitive inhibitor of Syk kinase and it might exert its anti-inflammatories through the Syk pathway. As depicted in Figure 2c, Vam3 is a polyphenol hydroxyl natural product. Compared with other Syk inhibitors which contain different amounts of N atoms, Vam3 owns a polyphenol hydroxyl scaffold with no N atoms. This might provide a new strategy to design novel Syk inhibitors. However, the solubility of Vam3 in water is poor. Structural changes on Vam3 to improve its solubility should not decrease the binding affinity of Vam3. Therefore, interaction between Vam3 and Syk interaction should be understood first. Indeed, characterizing the 3D-structure of Syk–Vam3 complex using crystallization or nuclear magnetic resonance (NMR) techniques is the best way, but it is time and resource consuming.

Fortunately, the comparably fast and inexpensive docking protocols can be combined with accurate but more expensive molecular dynamics (MD) simulation techniques to predict more reliable protein–ligand complex structures [19,20,21]. In our work, molecular docking and dynamics simulation were carried out to investigate the binding mode of the Vam3 with Syk. To investigate the reliability of our stimulation methods, OSB and 1B6 were employed as controls during the docking studies and dynamics simulations. Resveratrol, the monomer of Vam3, was used as a negative control to validate the binding mode of Vam3–Syk complex. We hope that we can reveal the mechanism of the Vam3–Syk interaction and give some useful information to structure optimization of Vam3 as Syk selective inhibitor with good properties.

## 2. Results and Discussion

### 2.1. Vam3 Inhibited Syk Kinase Activity in Vitro

Resveratrol is a polyphenolic compound found in grapes. Previous studies reported that resveratrol was a major Syk inhibitor and inhibits activation of Syk kinase in mast cell [22,23]. Vam3 is a derivative of resveratrol. Ring-C and D of Vam3 share the same structure with Resveratrol. This suggests that Vam3 may also have the capacity for Syk inhibition.

To confirm that Syk was the cellular target of Vam3, *in vitro* kinase assays were performed by using purified Syk protein. As shown in Figure 2, Vam3 inhibited Syk kinase activity with an IC_50_ of 62.95 nM and Vam3 was shown to be an ATP-competitive inhibitor of Syk kinase with a Ki of 61.09 nM.

### 2.2. Extra Precision Docking Studies

**Figure 3:**
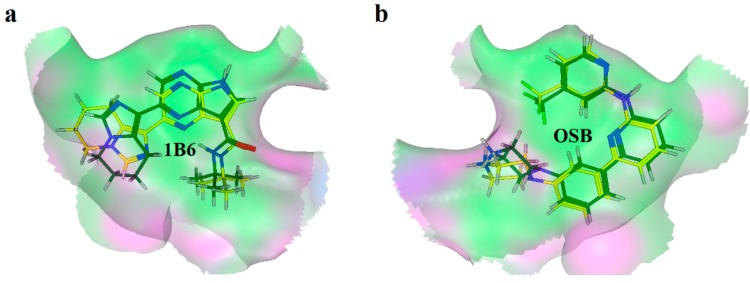
Superposition of conformations of docking results of 1B6 (**a**) and OSB (**b**).

**Figure 4:**
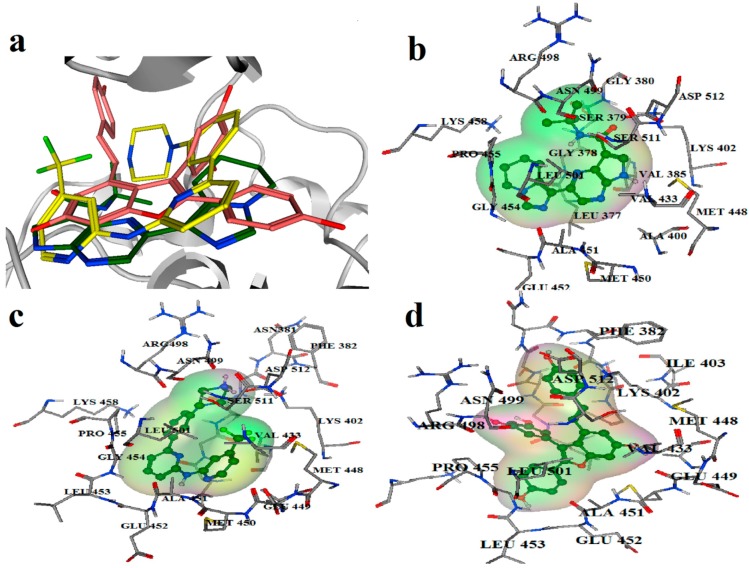
(**a**) Docked structures of 1B6 (green), OSB (yellow) and Vam3(pink) with Syk; (**b**) The binding site positioned around 1B6; (**c**) The binding site positioned around OSB; (**d**) The binding site positioned around Vam3.

Extra precision docking of Glide was carried out to investigate the binding mode of Vam3 with Syk. As for 1B6 and OSB, as revealed in Figure 3, two binding conformations of docking were performed respectively and there was no large difference between them. Therefore the conformations which achieved the highest GlideScore (G-score) were used as the initial structures for future binding mode analysis including a 15 ns MD simulation. As for Vam3, however, only one binding conformation was performed. This mainly came from the large rigidity of Vam3 and special shape of the ATP-binding pocket of Syk. Therefore the only credible docking result of Vam3 was used in future binding mode analysis. As shown in Figure 4, the three molecules (1B6, OSB and Vam3), as all of them are ATP-competitive inhibitor of Syk, were docked into the APT-binding pocket of Syk and all of them were positioned in the same location of Syk. 1B6 and OSB possessed a “U”-shaped conformations in the pocket while Vam3 shown the “ψ”-shape conformation. The binding modes of 1B6, OSB and Vam3 are shown in panels of Figure 4b–d, respectively. The detailed interactions will be discussed further in the following molecular dynamics simulations.

### 2.3. Molecular Dynamics Simulation Studies

**Figure 5:**
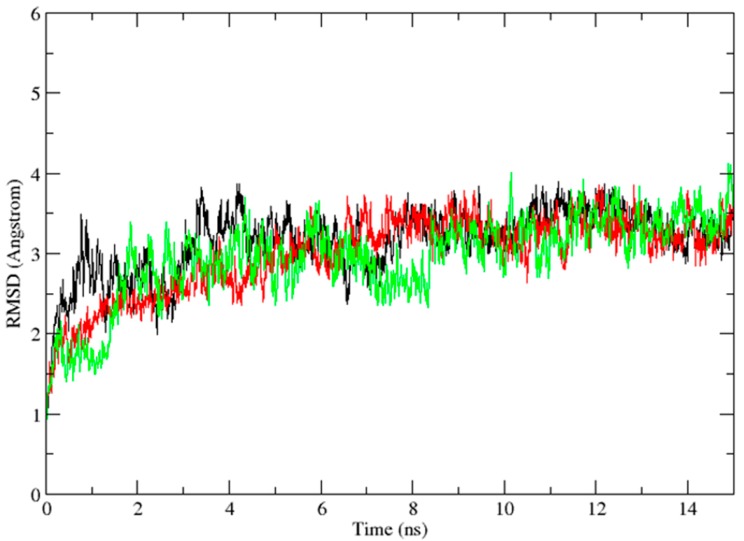
The results of molecular dynamics (MD) simulation. The MD simulation time *vs.* root mean-square deviation (RMSD) of the backbone atoms for 1B6-bound system (black), OSB-bound system (red) and Vam3-bound system (green).

In the docking studies, flexibility of the protein was not taken into consideration. In order to find the key residues and position of Vam3–Syk interaction, we performed 15 ns MD simulations with the Desmond program in which flexibility of proteins were taken into consideration. Three different systems were studied, including 1B6-bound system, OSB-bound system and Vam3-bound system. 1B6-bound system and OSB-bound system were taken as controls. The root mean square deviation (RMSD) values of the backbone atoms relative to the initial structure were calculated to measure the convergence of the systems and ensure the rationality of the sampling method. As depicted in Figure 5, the RMSD of the three were about 3.5 Å after 10 ns and all of them almost remained at this level in the following simulation processes. This indicated that the three systems were stable after 10 ns of simulation.

**Figure 6:**
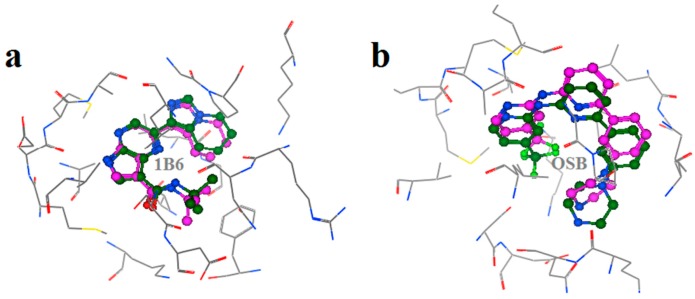
(**a**) Superposition of MD average structures of 1B6 with Syk (purple) and crystal structures of 1B6–Syk complex (green); (**b**) Superposition of MD average structures of OSB with Syk (purple) and crystal structures of OSB–Syk complex (green).

**Figure 7:**
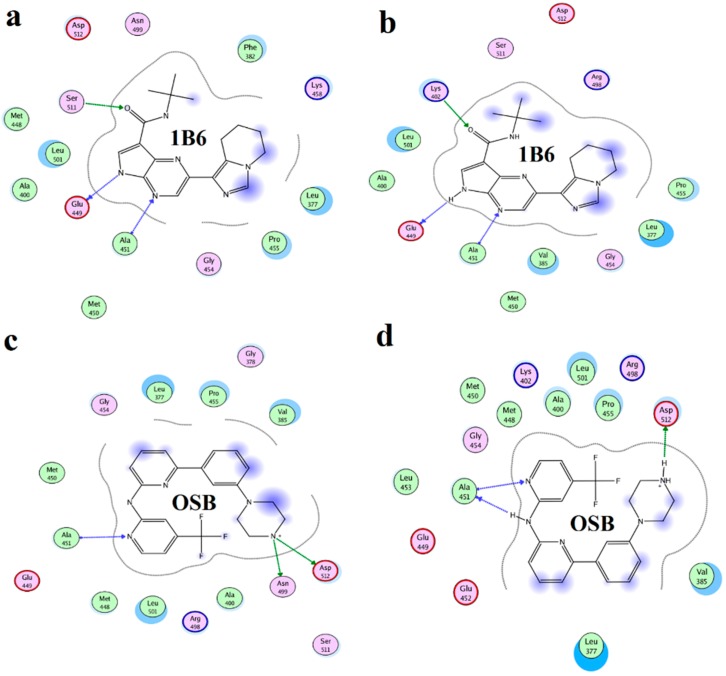
The key interactions between 1B6 and OSB with Syk in the crystal structures are shown in (**a**,**c**); The key interactions between 1B6 and OSB with Syk in the MD average structures are preformed in (**b**,**d**), respectively.

To estimate the difference between the MD average structures and crystal structures of controls, the average structures of the MD-simulated complexes from the last 5 ns of MD simulations were superimposed with the crystal structure of 1B6–Syk and OSB–Syk complexes. As shown in Figure 6 and Figure 7, the MD average structures of the two control complexes are overall very similar to their crystal structures. As for 1B6-bound system, 1B6 formed the same H-bonds with Glu449 and Ala451 of Syk just as in crystal structure. The same hydrophobic interactions with Leu377, Pro455 and Leu501 were also observed. Similarly in OSB-bound system, the same H-bonds and hydrophobic interactions in crystal structure of OSB with Syk were also found in MD average structure of OSB–Syk complex. However, there were also little difference between MD average structures and crystal structures of controls. For example, H-bond with Lys402 in the crystal structure of 1B6 with Syk did not exist in crystal structure of 1B6 with Syk. OSB formed an H-bond with ASN499 but it was not found in MD average structure of OSB–Syk complex. The little difference between the MD average structures and crystal structures come from the little location drift and the later might due to the reason that we used whole Syk in our stimulations while crystal structures only contains catalytic domain. These results suggested that our methods, which using docking and molecular dynamics stimulation to investigate the interaction between Vam3 and Syk was reasonable and the MD average structures were very similar with the crystal structures.

**Figure 8:**
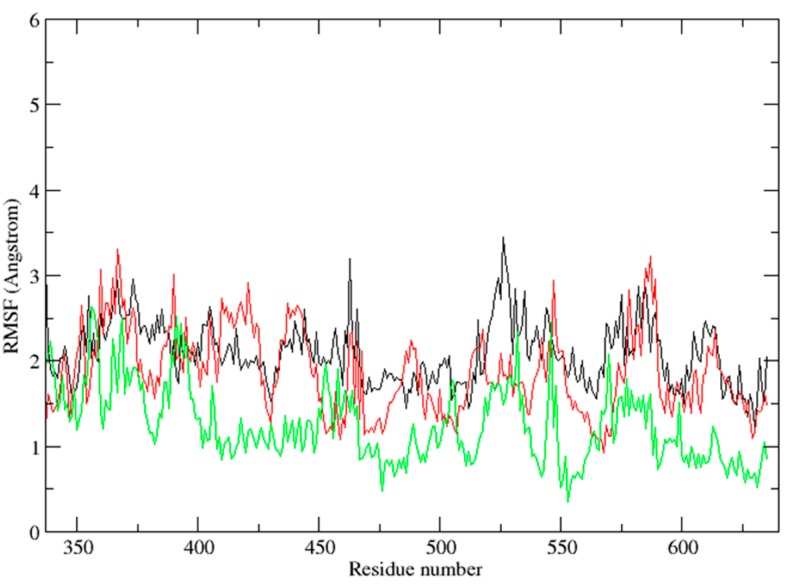
The root-mean-square fluctuation (RMSF) of the catalytic domain of Syk for each residue for the 1B6-bound (black), OSB-bound (red) and Vam3-bound (green) system.

Moreover, the analysis of root-mean-square fluctuation (RMSF) *vs.* the residue number for these three systems is illustrated in panel of Figure 8. In the figure, the ligand was not included. The residues (amino acids 377–628) of the catalytic domain was evaluated. RMSF can reflect the mobility of the residue around its mean position and is helpful to find the residues and regions of Syk with major conformational changes. Obviously, the RMSF distribution of Vam3-bound system was different with 1B6- and OSB-bound systems, which indicating that Vam3 could have a distinct interaction mode with Syk. This may come from the difference between the structures of Vam3, 1B6 and OSB.

### 2.4. Binding Mode Analysis of Vam3–Syk Complex

**Figure 9:**
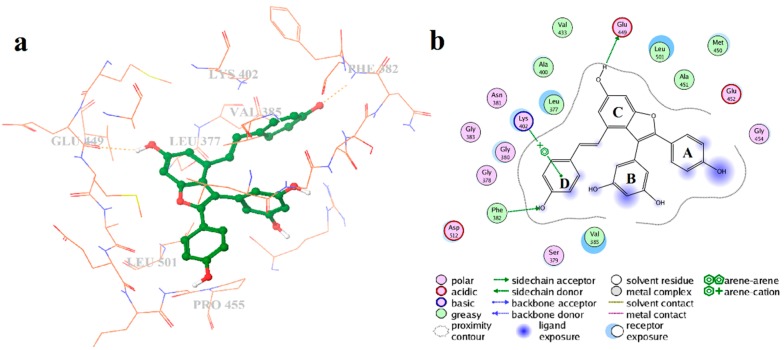
Binding mode of Vam3 with Syk. (**a**) The key Vam3–Syk interactions in the Syk ATP binding pocket in 3-dimensional structure; (**b**) The two-dimensional projection of Vam3-Syk interaction.

The binding mode for the MD average structures of Syk with vam3 is displayed in Figure 9a,b. It can be observed that Vam3 extend deeply into the binding site of Syk. As is revealed previously, compared to 1B6 and OSB of which both could form three H-bonds with Syk, Vam3 could form two H-bonds with Syk. The H atom on 11b-OH of Vam3 could form hydrogen bond with the backbone atom of Glu449 and this kind of H-bond was also observed in 1B6–Syk interaction. The O atom on 4b-OH of Vam3 also had hydrogen bond interaction with the backbone atom of Phe382 and it is not observed in 1B6–Syk and OSB–Syk interactions or other inhibitors–Syk interactions. However, Vam3 could not form H-bond with Ala451 and Asp512 which many Syk inhibitors favors to form H-bonds with. In addition, arene–cation action between ring-D of Vam3 and Lys402 was also observed. These results indicated that ring-C and ring-D and the two –OH groups on them were necessary for the activity of Vam3.

Hydrophobic interactions are also presented between Syk and Vam3. The phenyl ring-B of Vam3 formed hydrophobic interaction with the side chain of Val385 and ring-A formed hydrophobic interactions with the side chain of Leu377 and Leu501. Fernando Padilla *et al.* had demonstrated that optimizing interactions of ATP-competitive Syk inhibitors with Pro455 and Asn457, present in only nine aligned kinases of a total of 433, was an attractive way of introducing high levels of Syk specificity [12]. Unfortunately, interaction between Pro455 and Vam3 or Asn457 and Vam3 was not observed. This suggested that Vam3 may not have good Syk selectivity profile.

Furthermore, to validate the binding mode of Vam3 and Syk, the same molecular docking and 15 ns MD simulation studies of resveratrol and Syk was carried out. Resveratrol is the monomer of Vam3. It also possesses the same polyphenol hydroxyl structure like Vam3 and shows weak potency for Syk inhibition. Therefore, molecular docking and MD simulation studies on resveratrol may help us better understand the binding mode of Vam3 and Syk.

**Figure 10:**
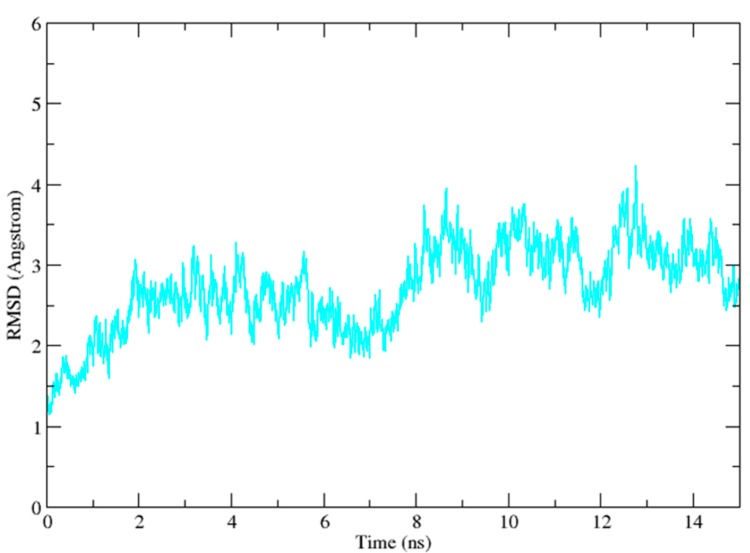
The MD simulation time *vs.* root mean-square deviation (RMSD) of the backbone atoms for resveratrol–Syk system.

Resveratrol was docked into the ATP-binding pocket of Syk and the conformation with the higher G-score was used in the MD simulation study. As depicted in Figure 10, the RMSD of the resveratrol–Syk system had reached a plateau after 8 ns. There were RMSD fluctuations from 10 to 15 ns as expected, and the fluctuations may come from the small size and weak binding ability of resveratrol. This indicated that the simulation was not necessarily converged when it reached a plateau in the RMSD.

**Figure 11:**
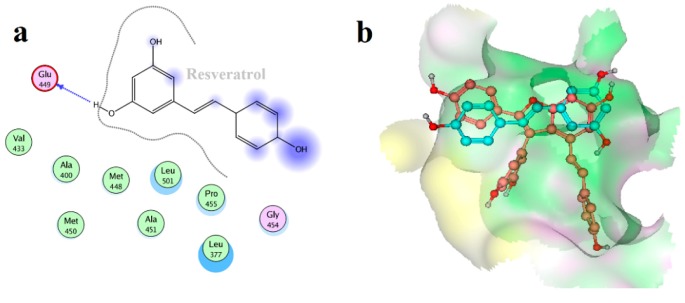
(**a**) The two-dimensional projection of resveratrol–Syk interaction. (**b**) Superposition of MD average structures of resveratrol with Syk (cyan) and Vam3 with Syk (pink).

**Figure 12:**
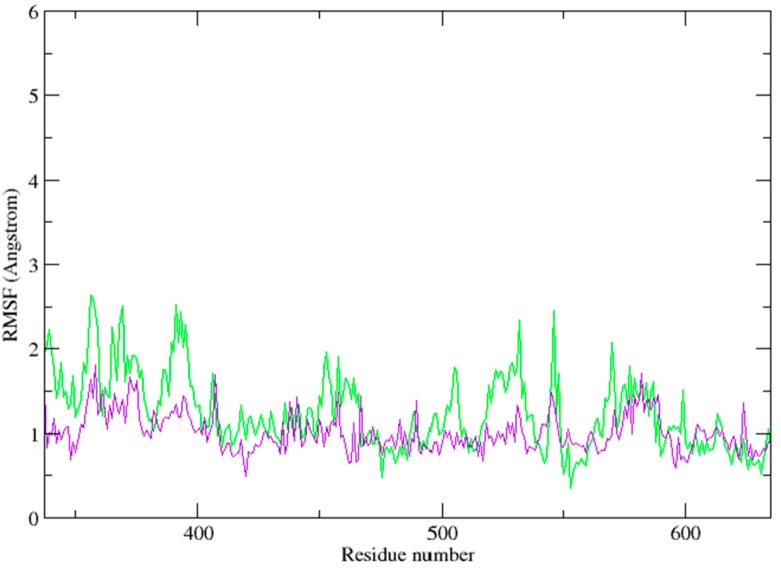
The RMSF of the catalytic domain of Syk for resveratrol-bound system (purple) and Vam3-bound system (green).

The average structure of resveratrol–Syk complex from the last 5 ns of the MD simulation was carried out, as shown in Figure 11a. As for resveratrol, it formed H-bond with Glu 449 of Syk and hydrophobic interactions with Leu 377 and Leu 501. As revealed in Figure 11b, resveratrol positioned the same location with ring-A and C of Vam3. Similarly, Vam3 could formed H-bond with Glu 449 of Syk and hydrophobic interactions with Leu 377 and Leu 501. Besides, ring-D of Vam3 formed another H-bond with Phe 382 of Syk and arene–cation action with Lys 402 of Syk. This could explain the potency for Syk inhibition of Vam3 is much better than resveratrol. In addition, to compare the binding modes of Vam3 and resveratrol with Syk, the RMSF analysis was carried out. As depicted in Figure 12, the RMSF distribution of resveratrol-bound system was similar with Vam3-bound system. This may come for the similar structures between resveratrol and Vam3. In summary, resveratrol and Vam3 which have the same skeleton share the similar inhibition mechanism for Syk. More interactions were observed between Vam3 and Syk than resveratrol, which could explain the better inhibiting capacity for Syk of Vam3. These evidence suggest that the binding mode of Vam3–Syk complex is reasonable.

Therefore, as the essential roles of ring-C and D in Vam3–Syk interaction, structural optimization of Vam3 could focus on ring-A and B of Vam3 using chemical approach, such as bioisosteres [24]. In addition, optimization of ring-A, the hydrophobic ring, with hydrophilic groups may improve its solubility.

## 3. Materials and Methods

### 3.1. Experimental Studies

#### 3.1.1. Plant Material

Vam3 was isolated from the ethanol extracts of a methanol extracts of *Vitis amurensis* Rupr, as described previously. This compound was prepared by dissolving in dimethyl sulfoxide (DMSO) and the final concentration of DMSO was adjusted to 0.1% (*v*/*v*) in culture media.

#### 3.1.2. *In Vitro* Fluorescence Polarization Kinase Assay

The reactions were carried out in a total volume of 25 μL in 96-well microtiter plates. The Syk tyrosine kinase activity at single dose concentration of 12.5 ng/μL, 10 μL of volume, was carried out served as the enzyme source. The total volume of 10 μL mixture containing 0.2 μg/μL Poly (Glu, Tyr) sodium salt (4:1, Glu:Tyr, Sigma–Aldrich, St. Louis, MO, USA) and 10 μM ATP (Promega, Madison, WI, USA) served as the standardized substrate. The concentration range of the tested inhibitors employed in reactions was 0.0032, 0.016, 0.08, 0.4, 2, 10 μM or DMSO with 5 μL volume. All of the enzymatic reactions were conducted at 37 °C for 60 min. The assay was terminated by adding 25 μL of ADP-GloTM Reagent (Promega, Madison, WI, USA). The 96-well plate was shaken and then incubated for 40 min at ambient temperature. Fifty microliter of Kinase detection reagent was added and the 96-well reaction plate was then read using the ADP-Glo Luminescences Protocol on a GloMax plate reader (Promega: Catalog #E7031). For each concentration of Vam3, the rate of reaction at each concentration of ATP was determined and plotted against the ATP concentration to determine the apparent *K*_m_ and *V*_max_ (maximal rate). Finally, the apparent *K*_m_ (or apparent *K*_m_/*V*_max_) was plotted against the inhibitor concentration to determine the *K*_i_. All data analysis was performed using Prism and Prism enzyme kinetics programs.

### 3.2. Computational Studies

#### 3.2.1. Preparation of Protein Target Structure

The crystal structure of full-length Syk in complex with ANP (PDB code: 4FL2, with the resolution of 2.19 Ǻ) was retrieved from the RCSB Brookhaven Protein Data Bank (PDB) [6]. The structure is the only well-defined full-length Syk with a resolution of 2.19 Ǻ except for the first part of the *N*-terminus (amino acids 1–8) and for the interdomain linker region (amino acids 265–336). Indeed, using the catalytic domain alone in the simulation would save time. However, the simulation of the whole protein with inhibitor would provide more information about the SH2 domains, which is helpful to further study. Therefore 4FL2 was used as the receptor. Furthermore, 4FL2 is a full-length Syk in complex with AMP–PNP revealing an autoinhibited conformation which is close to the conformation of Syk–ATP complex. Therefore, using 4FL2 as the receptor is more suitable than the other structures and can make docking and MD simulation results persuasive and convincing. As for the missing residues being far away from the ATP-binding pocket, lacking of these 73 residues would not largely affect the results of our simulations. Therefore, we do not model this part. Then the structure was prepared using the following procedures by the Protein Preparation Wizard in the Schrödinger software suite, including adding hydrogen atoms, assigning partial charges using the OPLS_2005 force field and assigning protonation states, and structure minimizing in vacuum. Finally, the cocrystal ANP was removed, and the resulting structure was used as the receptor model in the following studies.

#### 3.2.2. Ligand Preparation

The structure of Vam3 and resveratrol was constructed using Mastro [25], while the ligands OSB and 1B6 were retrieved from the Protein Data Bank (PDB code: 4F4P and 4Y0T, respectively) [10,12]. All the ligands were prepared by using the LigPrep [26] and then to proceed with stereoisomer generation, neutralization of charged structures and determination of the most probable ionization state at pH 7.2 ± 0.2. The OPLS-2005 forcefield was used for optimization to produce the low-energy conformer of the ligand [27].

#### 3.2.3. Molecular Docking

The ligands Vam3, resveratrol, OSB and 1B6 were docked into the receptor using Glide software [28]. Glide approximated a complete systematic search of the conformational, orientational and positional space of the docked ligand, and a series of hierarchical filters was used to search for possible locations of the ligand in the active-site region. In this work, grid box was centered on the ATP centroid in the X-ray crystal structure of Syk and the ligands were docked into the box using the “extra precision” glide docking (Glide XP) which docks ligands flexibly and the protein rigidly. The quality of the geometric matches of the docked binding structures with the lowest GlideScore was visually checked and the best one was selected as the initial complex for further studies. GlideScore is based on ChemScore, but includes a steric-clash term and adds buried polar terms devised by Schrödinger to penalize electrostatic mismatches:

GScore = 0.065 × vdW + 0.130 × Coul + Lipo + H-bond + Metal + BuryP + RotB + Site (1)

where vdW, Coul, Lipo, H-bond, Metal, BuryP, RotB and Site are the van der Waals energy, Coulomb energy, Lipophilic contact term, Hydrogen-bonding term, Metal-binding term, Penalty for buried polar groups, Penalty for freezing rotatable bonds and Polar interactions in the active site, respectively.

#### 3.2.4. Molecular Dynamics Stimulation

The initial coordinates for the MD calculations were taken from the docking results. For each system, MD studies were performed using OPLS_2005 force field in an explicit solvent with the TIP3P model [29] of water within the Desmond software. The dimensions of each orthorhombic water box were 100 × 100 × 100 Å, which ensured that the entire surface of each complex was covered by the solvent model and the systems were neutralized by adding Cl^−^ counter ions to balance the net charges of the systems. Before equilibration and long production MD simulations, the systems were minimized and pre-equilibrated using the default relaxation routine implemented in Desmond [30]. The solvated system was minimized first with solute restrained and then again minimized without solute restraints by using hybrid method of steepest descent and the LBFGS (limited-memory Broyden–Fletcher–Goldfarb–Shanno) algorithm with a maximum of 2000 steps including initial 10 steps of steepest descent. The minimized system was passed through a short 12 ps simulation in the NVT ensemble using a temperature of 10 K with nonhydrogen solute atoms restrained. Subsequently, the system was simulated for 12 ps in the NPT ensemble using temperature 10 K with restraints on nonhydrogen solute atoms. In the next step, the system was simulated for 24 ps in NPT ensemble using a temperature of 300 K restraining the nonhydrogen solute atoms. In the last step of equilibration process, the system was further simulated for 24 ps in the NPT ensemble with no restraints at temperature 300 K. The temperatures and pressures in the short initial simulations were controlled using Berendsen thermostats and barostats, respectively. Then, each system was performed for a 15 ns long production MD simulation. The OPLS_2005 force field was used along with the MacroModel module [31] to provide and check the necessary force field parameters for the ligands. When MacroModel performs an energy calculation, the program checks the quality of each parameter in use. The use of low quality parameters, especially torsional ones, may result in inaccurate conformational energy differences and geometries. Bond, angle, torsional angle and improper angle checked parameters were listed as high- and medium-quality force field parameters for all ligands studied. During the MD simulations, the equations of motion were integrated with a 2 fs time step in the NPTensemble. The Shake algorithm was applied to all hydrogen atoms; the van der Waals (VDW) cutoff was set to 9 Å [32]. The temperature was maintained at 300 K, employing the Nose-Hoover thermostat method with a relaxation time of 1 ps [33]. Long-range electrostatic forces were taken into account by means of the particle-mesh Ewald (PME) approach [34]. Data were collected every 12 ps during the MD runs. Visualization of protein–ligand complexes and MD trajectory analyses were carried out with the VMD software package [35,36]. The equilibration was monitored by examining the stability of the temperature, energy, and the density of the system as well as the RMSD of the backbone atoms.

## 4. Conclusions

In this study, we first demonstrated that Vam3 is an ATP-competitive inhibitor of Syk with IC_50_ of 62.95 nM and Ki of 61.09 nM by using *in vitro* fluorescence polarization kinase assay. Moreover, to investigate the mechanism of Vam3–Syk interaction, docking studies and molecular dynamics stimulations were performed. Through our stimulations, we have predicted optimal binding conformation of Vam3 with Syk. 11b-OH and 4b-OH of Vam3 formed two H-bonds with Glu449 and Phe382 in the active site of Syk, respectively. Arene-cation action was also found in Vam3–Syk interaction. Together with hydrophobic interactions, these actions form the basis of the well inhibitory activity of Vam3. These results may not only useful for the structural optimization of Vam3 but also for the rational design of novel Syk inhibitors with new scaffold.
